# Citizen Debates in Social Networks about Didactic Resources for Mathematics

**DOI:** 10.3390/ijerph182111686

**Published:** 2021-11-07

**Authors:** Rosa Valls-Carol, Garazi Álvarez-Guerrero, Garazi López de Aguileta, Álvaro Alonso, Marta Soler-Gallart

**Affiliations:** 1 Department of Theory and History of Education, University of Barcelona, 08035 Barcelona, Spain; rosavalls@ub.edu; 2 Faculty of Psychology and Education, University of Deusto, 48007 Bilbo, Spain; garazialvarez@deusto.es; 3 Department of Curriculum & Instruction, University of Wisconsin-Madison, Madison, WI 53706, USA; lopezdeaguil@wisc.edu; 4 Department of Sociology, UNED, 28040 Madrid, Spain or alvaroalonsolamilla@gmail.com; 5 Department of Sociology, University of Barcelona, 08034 Barcelona, Spain

**Keywords:** citizens, social media analytics, mathematics, didactic resources, scientific evidence, social impact

## Abstract

Citizens are increasingly turning to social media to open up debates on issues of utmost importance, such as health or education. When analyzing citizens’ social media interactions on COVID-19, research has underlined the importance of sharing and spreading information based on scientific evidence rather than on fake news. However, whether and how citizens’ interactions in the field of education, particularly in mathematics, are based on scientific evidence remains underexplored. To contribute to filling this gap, this article presents an analysis of citizen debates in social networks about didactic resources for mathematics. Through social media analytics, 136,964 posts were extracted from Reddit, Instagram, Twitter and Facebook, of which 1755 were analyzed. Results show that out of the 213 posts of citizen debates on didactic resources for mathematics, only two contained scientific evidence and eight claimed to contain scientific evidence. These findings highlight the importance of promoting actions to encourage citizen debates around didactic resources for mathematics based on scientific evidence.

## 1. Introduction

Today, as a result of the dialogic turn in society and in science [1,2], research projects funded by the European Commission are required to co-create knowledge with citizens by including their voices throughout the whole research, and to provide evidence of their social impact or the potential to achieve it in the future [3,4]. These two requirements go hand in hand in the advancement of a science at the service of citizens that responds directly and urgently to their needs, which citizens themselves are increasingly demanding [5,6]. However, if we want to contribute to improving human lives, it is not enough for us to co-create knowledge on how to improve them; we need to make such knowledge and evidence available and accessible to them [7]. In other words, we need to engage citizens in scientific research.

Along this line, research has stressed the importance of investigating the dialogues between science and society in order to fathom the social improvements science is generating and, thus, promote citizens’ engagement with it [8]. Social networks are becoming an optimum space for investigating these dialogues. Indeed, more and more people are turning to social networks seeking and discussing scientific evidence. This can be clearly seen in the field of health [9], where citizens often engage in social networks to share and look for scientific evidence regarding different health issues. In this vein, analyzing how individuals interact with each other in social networks has been shown to be important for understanding, for example, vaccine refusal, which can give useful information for effective health communication campaigns [10]. Analyzing social media also helps include the data of those usually excluded from traditional samplings (such as random-digit dialing or address-based sampling), as social networks such as Twitter have been shown to be very popular among members of vulnerable groups [10]. However, some research has also warned that citizens are often exposed to information related to health lacking scientific evidence [9].

Now more than ever, the COVID-19 pandemic has drawn attention to the spread of information (or misinformation) in social networks. Citizens have used social media to obtain information on essential topics such as the novel COVID-19 virus, face masks, disinfectant or the symptoms caused by the virus [11]. Although much research has focused on the spread of false news about the virus, scientific investigations have also shown that many interactions related to the pandemic in social media contained veracious information, rather than misinformation. Concretely, research by Pulido and colleagues [12] showed that, whereas false information was tweeted more, it was less likely to receive retweets. In turn, tweets containing scientific evidence or fact-checking information were more likely to be retweeted. The higher presence of interactions containing evidence and not false news has also occurred with other health topics [13]. Moreover, there is also evidence that the misperceptions some people have due to misinformation or false information in social networks can be changed when other users correct them by providing scientific evidence to support the argument [14].

In short, as more and more people are turning to social networks to discuss, share or seek information about health-related issues, more and more research is being focused on what those tweets, posts and other social media interactions look like. To a lesser degree, the interactions that citizens are having in social networks about education-related issues are also increasingly being studied. For instance, scientific literature has evidenced that some teachers who participate in social media share resources among them as a means of professional learning [15]. In this vein, there is an increasing line of investigation about technology and mathematics. Many mathematics teachers seek resources in social media (such as Pinterest) to implement in their classrooms [16]. Self-administered Facebook groups of mathematics teachers also serve as a tool for sharing pedagogical knowledge and materials or methods applied in their classes [17]. Other research has focused on the improvement of linguistic abilities of mathematics teachers through online courses [18]. However, not all those resources chosen by mathematics teachers improve students’ achievements, as some of them even worsen them [16]. This can have devastating consequences for students, as poor mathematics achievement is linked to higher rates of school dropout and failure [19].

For teachers and other citizens to use mathematics resources that help improve students’ achievements and learning, such resources need to be based on scientific evidence of social impact, that is, there needs to be scientific evidence that shows the improvements linked to using those resources. The project *ALLINTERACT, Widening and diversifying citizen engagement in science*, founded by the European Commission’s Horizon 2020 Framework Programme, is currently being developed with the aim of shedding light on how citizens currently or potentially interact with scientific evidence in order to promote their participation with science, thus contributing to all citizens’ human right to science. Running from October 2020 to May 2023, researchers from 16 institutions throughout Europe are and will be contributing new knowledge on how to promote citizens’ engagement in scientific evidence on two particular Sustainable Development Goals: Quality Education and Gender Equality. In spite of being of utmost relevance for citizens, more efforts are needed to make them aware of the social impact of research on education and gender in order to promote their engagement with it. Parents, teachers and students need to know, among other things, that there are currently thousands of schools implementing Successful Educational Actions based on scientific evidence of the social impact that are fostering the educational success of all students, including the most vulnerable such as the Roma community, in mathematics [20]. Knowing what the educational actions and resources are that have been scientifically proven to achieve the best results allows parents, teachers, students and citizens in general to make scientifically informed decisions that will improve all students’ learning and lives.

The increasing citizen debates on social networks around topics such as education, as well as the increasing accessibility and openness of scientific evidence, leave great potential for enhancing citizens’ engagement with scientific developments in mathematics in social networks. Although sharing information in social networks often entails the risk that many users will post or share content that contains false information, there are some initiatives and studies focused on promoting the sharing of information based on scientific evidence. Such initiatives, following the European Commission’s line on co-creation promoted in the current Horizon Europe Framework Programme, aim at engaging diverse citizens and scientists in dialogues in order to co-create scientific evidence and distinguish them from hoax or misinformation, promoting the spread of evidence among social networks. An example would be the Adhyayana and Sappho Scientific Evidence Platforms, the former based on education and the latter on gender. In these science-based participatory tools, developed from a bottom-up approach, researchers, practitioners and citizens engage in an egalitarian dialogue based on validity claims, that is, that any person can make any claim as long as this is supported by either scientific evidence or by arguments from everyday experiences. After 14 days, the platform evaluates whether such a post contains scientific evidence and categorizes it either as hoax or as evidence. Such bottom-up approaches, therefore, provide citizens with more knowledge and arguments to be able to distinguish when information shared in social networks is based on misinformation or on scientific evidence. Hence, while these initiatives do not overcome the use of misinformation in social networks, they provide social network users with more knowledge, arguments and tools to be able to promote the spread of information containing scientific evidence and to distinguish it from and disregard false information.

However, in order to develop more actions and programs such as the aforementioned, scientists need further analyses that will allow us to know whether or not there are citizens already engaging in debates on mathematics based on scientific evidence. This area of research is still underexplored. To contribute to it, the present study explores whether and how citizen debates in social networks about didactic resources for mathematics are based on scientific evidence.

## 2. Materials and Methods

### 2.1. Research Design

**Figure 1:**
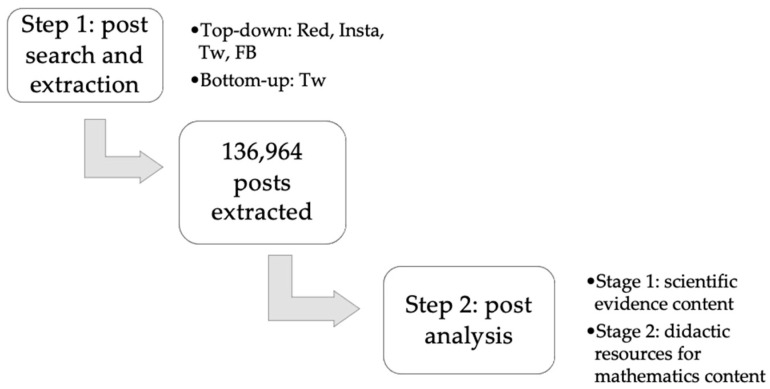
Design and analysis process.

The present research has been developed following the communicative approach [21,22,23], which focuses not only on the analysis of reality but also in identifying and including citizens’ and participants’ voices to identify possible pathways towards social transformation. In this vein, following the increasing trend of citizens turning to social networks, analyzing their interactions in such networks offers researchers the possibility to grasp citizens’ voices about essential issues in their everyday lives and society. Indeed, more and more researchers are collecting data through social media, as it has proven to be a way of including citizens’ voices in issues of relevance [24]. Thus, and in line with the objective of the present study, a social media analysis (SMA) [24] was carried out in Reddit, Instagram, Twitter and Facebook by researchers from 6 different institutions. SMA methodology aims at collecting citizens’ voices in social media networks, it helps identify the needs and concerns of citizens, and is later useful for redefining our own research goals [24]. These four social networks were selected due to their presence and use by individuals all over the world. All four chosen social networks are among the world’s most used 17 social networks, with Facebook the most used in the world with 2853 million users. Instagram has 1386 million, Reddit 430 million and Twitter 397 million users [25]. Figure 1 summarizes the design and analysis process.

First, 136,964 posts were extracted through searching hashtags (in Instagram and Twitter), pages (in Facebook) and communities (in Reddit). Criteria for selecting hashtags on Instagram and Twitter were based on (a) their popularity, (b) the correspondence with the topics of the ALLINTERACT project, (c) the level of interactions among citizens in them and (d) the presence of citizens in the posts. Regarding Facebook pages, selection criteria corresponded to (a) a high level of activity and interaction, (b) outreach of the page and (c) the type of content shared (such as presenting scientific evidence). Last, the selected Reddit communities met the criteria of (a) having interactions through debate, (b) having a high outreach and level of interactions and (c) including topics related to the ALLINTERACT project.

The extraction of data was conducted following the two approaches that characterize SMA, as defined by Cabré-Olivé and colleagues [24]. On the one hand, following the top-down strategy, researchers first dialogically defined keywords related to the topics of the ALLINTERACT project and then contrasted how these were expressed by citizens in the selected social networks. The top-down approach was used in all four social networks. On the other hand, the bottom-up strategy was also included in Twitter. This approach consists of identifying the most used keywords and hashtags and then contrasting them with the topics of the project. Using both strategies allows to both contrast the topics of the project with the opinions of individuals in social media (through the top-down strategy) and to identify topics emerging from users’ interactions that have not yet been covered.

All the social media data were extracted through NVivo and Python. The chosen hashtags, pages and subreddits in each social network are presented in Table 1, Table 2, Table 3 and Table 4 together with the number of posts extracted for each one.

### 2.2. Data Analysis

Following the postulates of the communicative approach, a communicative content analysis [12] was conducted. Thus, all the analysis was based on an in-depth dialogic process, where researchers engaged in dialogue on the classification of each content.

The analysis was conducted in two stages. In the first stage, the scientific content, or lack thereof, of all the posts extracted was analyzed. To that end, three main categories were established according to the scientific sources of each post: 0 not scientific evidence (when the post did not contain scientific evidence); 1 certified scientific evidence (when the post included articles from journals indexed in Scopus and/or JCR); 2 supposed scientific evidence (when the post claimed to be based on scientific evidence but no reference to the study or article was made). In the cases where a link was provided in the post, the link was also included in the unit of analysis.

All the posts were analyzed by pairs of researchers. The first researcher assigned a number depending on the scientific sources of the posts (0–2), and the second researcher double checked the analysis already made. When the two researchers did not share the same view, they dialogically discussed and reached an agreement to categorize the post. This criteria definition and the analysis have been developed by researchers from 6 different institutions.

At the second stage, the analysis focused on whether the posts’ content was related to didactic resources for mathematics or not. To that end, researchers dialogically defined 7 keywords related to didactic resources for mathematics: calculation, numbers, numeracy, numeracy skills, manipulatives, resources and math. A total of 3555 posts were extracted through the keyword search, of which 1800 were reposts or posts appearing more than once with different keywords. All posts found for each of the keywords were read through several times by researchers. As a result of this analysis, those posts that included content related to mathematics didactic resources were selected first. After reading those selected posts again several times, a number of posts were discharged because they were used for marketing of some mathematics didactic resources, rather than being citizens talking about or discussing them. Table 5 summarizes the number of posts found with each keyword in each social network analyzed.

### 2.3. Ethics

In order to grant anonymity of the users whose posts comprise the sample of this study, researchers will not disclose any personal information about the users, nor will they quote any post literally. Moreover, for this manuscript, Facebook pages and Reddit communities have been anonymized. In addition, when conducting the analysis, researchers did not enter into the profiles of the users who made the posts. All data have been anonymized and fulfilled the Regulation (EU) 2016/6791, the EU General Data Protection Regulation (GDPR) and the Terms and Conditions of each social network. The study was approved by the Ethics Committee of the University of Barcelona.

## 3. Results

In this section we present the results of the analysis of those posts that fit the inclusion criteria. We will first show the qualitative results of this analysis, following with a sub-section on the qualitative results achieved.

### 3.1. Quantitative Results

Results reveal that out of the 1755 posts analyzed, 213 contain interactions related to didactic resources for mathematics. The social network with most posts related to didactic resources for mathematics is Facebook, with 139 posts (65.2%). The second social network with most posts related to didactic resources for mathematics is Reddit, with 43 posts (20.1%), followed by Twitter with 23 posts (10.7%). Last, the social network where the least posts related to didactic resources for mathematics were found was Instagram, with 8 posts (3.7%).

**Figure 2:**
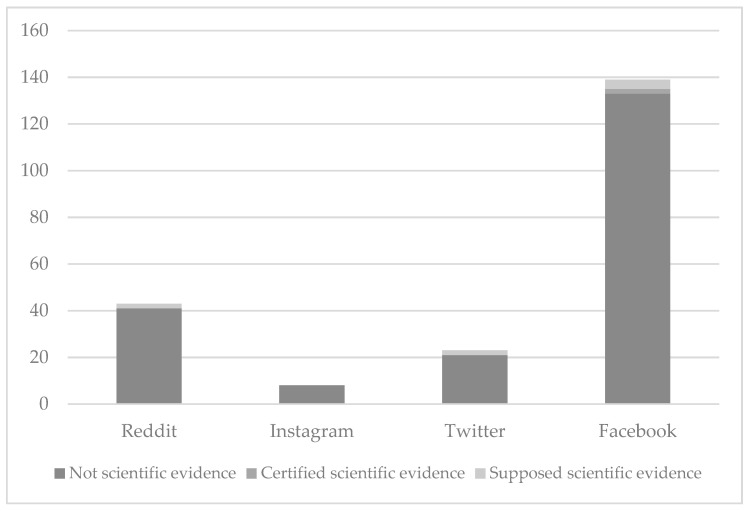
Number of posts related to didactic resources for mathematics per category in each social network analyzed.

Regarding the scientific evidence classification, the category in which most posts were found was the “not scientific evidence” category, with 203 posts (95.3%) in total. In contrast, the category in which the least posts were found was the “certified scientific evidence” category, with 2 (0.9%) posts belonging to this one. Last, 8 posts (3.7%) were found in the “supposed scientific evidence” category. Table 6 and Figure 2 break down all posts related to didactic resources for mathematics per category in each of the four social networks analyzed.

As the table shows, Facebook is the social network with most posts related to didactic resources for mathematics that claim to contain scientific evidence, with four posts in total (1.8%). It is also the only one with a post about didactic resources for mathematics that includes certified scientific evidence, having two in total (0.9%). Reddit and Twitter have the same number of posts related to didactic resources for mathematics claiming to contain scientific evidence, with two each (0.9%). Last, Instagram is the only social network in which no post about didactic resources for mathematics has been found to include certified nor supposed scientific evidence.

### 3.2. Qualitative Results

Whereas those posts categorized as containing certified scientific evidence or supposed scientific evidence were the least among all posts related to didactic resources for mathematics in the social networks analyzed, a diversity of interactions and users can be found in them in terms of what types of resources they share and debate. Although researchers have not viewed the profiles of any of the posts analyzed, in some of those posts users reveal information about themselves, such as saying they are teachers, or they are parents who want to teach their children, or need to study math-related content.

The two posts categorized as containing scientific evidence are framed within a Facebook debate in which different users are engaged in a discussion about whether or not children should use their fingers when conducting math problems. Whereas many of the users contributing to this discussion are teachers, the two posts marked as containing scientific evidence do not provide information about the users. The two share the same article from a scientific dissemination journal that provides arguments and scientific evidence supporting the idea that children should use their fingers in math classes. One of the posts only contains the link of the article, whereas the other includes one of the authors of the article and directly addresses another Facebook user in their post.

In addition, one of the posts categorized as containing supposed evidence is also engaged in the debate about using fingers. In that case, the user does not provide any link to scientific evidence, but he or she refers to a book by the same author of the article shared in the other two posts and provides a quote supporting the argument in favor of finger counting.

Other posts categorized as containing supposed evidence also refer to different pedagogies or learning strategies for mathematics. Two of them explicitly mention scientific research, although they do not provide any link or source to such research. One of them explains that research shows students need to be scaffolded when they do not understand math-related content, and the other talks about a particular mathematics curriculum based on research. Others do not explicitly mention research in the post, but they do provide links to articles that do talk about research—which, in turn, do not include evidence from articles indexed in JCR and/or Scopus.

The remaining posts categorized as containing supposed evidence provide links to different blogs or webpages where resources for teaching and/or learning mathematics can be found. Again, the posts themselves do not talk about scientific research. However, the websites or blogs linked in the posts do talk about scientific research, although they do not include scientific articles indexed in JCR and/or Scopus. For example, a user shares a newspaper’s learning resources, which includes different resources that teachers can use in their classrooms. Yet not all of those resources are based on research or, at least, it is not specified that they are based on articles published in JCR and/or Scopus journals.

Regarding those citizen debates that have been categorized as not containing scientific evidence, a variety of resources and ways in which those resources are shared and/or discussed can be found. Some users share books, webpages, free online repositories, blogs, apps or other social networks in which math resources can be found. Some of these posts respond to other users’ posts in which they ask for resources to learn or teach a variety of mathematics-related knowledge. In other cases, users share with their followers or potential viewers mathematics resources they find useful as teachers or learners.

Similar to some of those posts categorized as containing supposed scientific evidence, many posts categorized as not containing scientific evidence are posts in which users share techniques, games or activities for learning or teaching mathematics with other users. In some cases, users share videos—either made by themselves or which they have found on the internet—teaching, for instance, how to solve math problems or how to understand math concepts. In other posts, teachers or parents share math-related activities and games they have undertaken with children, such as using chocolate bars to teach them fractions, integrating mathematics concepts into arts classes or playing with cards. Other posts contain advice that teachers share about specific pedagogical strategies to help children understand math-related content or how to solve mathematical problems, such as scaffolding children’s problem solving.

In the same line as the two posts categorized as containing scientific evidence, some posts categorized as not containing scientific evidence revolved around discussions about pedagogies or strategies, such as using fingers or not, memorization, or using the calculator, among others. Whereas the large majority of posts engaged in this debate do not include nor mention scientific evidence, one of the users asks what the scientific literature says about using fingers versus memorization.

## 4. Discussion

To improve citizens’ lives, researchers need not only bring their voices and experience to the research we are conducting; we also need to bring the evidence we are co-creating with them to them [2]. In light of the evidence on the increasing trends of citizens discussing issues that are paramount to their lives, more and more research is looking at whether those debates incorporate a discourse of scientific advancements and knowledge to support their claims. The COVID-19 pandemic has bolstered debates in social media around health, as well as the use and spread of scientific evidence in such debates [12,13]. When it comes to debates in social networks on education in general, and mathematics education in particular, still little research has explored whether such debates contain scientific evidence or not. This study aimed at advancing in this direction by analyzing citizen debates in social networks about didactic resources for mathematics. Findings reveal that, although most debates analyzed do not contain scientific evidence, some social network users are already introducing scientific evidence when engaging in debates about didactic resources for mathematics.

Following existing research on teachers’ use of social media to share and seek didactic and pedagogic resources, findings from this study confirm that several users in the social networks analyzed do engage in debates about didactic resources for mathematics. Although this study did not inquire into the users’ profiles due to privacy issues, the posts analyzed allowed researchers to see a variety of users, from school teachers to parents to students at different grades, and other citizens. Therefore, this study contributes evidence that there are citizens with diverse connections to education, not only teachers, who are turning to social networks to talk about their concerns or questions, to seek help or to offer help and advice on didactic resources for mathematics. However, as previous studies have highlighted, not all mathematics resources shared on social media improve students’ results [16]. This might be due to the fact that those mathematics resources are not based on scientific evidence. When classroom practice in mathematics—as well as any other subject—is not based on scientific evidence, not only do students’ learning and academic achievements not improve, but it has devastating consequences for them, especially for the most vulnerable students [19]. Hence, it is essential for researchers to engage citizens with scientific evidence on education in order to promote their use and sharing of didactic resources for mathematics that are based on scientific evidence.

Now, in order to promote citizens’ engagement with scientific evidence on mathematics, researchers need to know whether and how citizens are using and/or talking about it. This study has advanced a step in this direction by analyzing whether and how citizens use scientific evidence in debates about didactic resources for mathematics in social networks. Through conducting SMA in Reddit, Instagram, Twitter and Facebook, this study has found that there are posts about didactic resources for mathematics that either include some reference to scientific research or claim to be based on scientific research. Granted, posts that contained certified or supposed scientific evidence were the least among the categories analyzed. However, the fact that there are users who already introduce scientific evidence when talking about didactic resources for mathematics in social networks points out that some citizens are beginning to incorporate a discourse of scientific evidence in social networks when talking about education-related issues. Even those cases in which users claim to introduce scientific evidence, or in which they ask what the scientific literature says about a particular issue in didactic resources for mathematics, these findings indicate that there is a concern among some citizens regarding what the scientific research on mathematics-related issues says, and that they turn to social media to talk about or share that concern. Based on these findings, future research should explore new ways and pathways to foment more social network users to engage in a discourse of scientific evidence when discussing didactic resources for mathematics.

The limitations of the present research must be addressed. First, although the social networks analyzed are among the 17 most popular in the world, other highly used networks such as TikTok or Sina Weibo have not been included. In spite of having analyzed diverse networks, the inclusion of other social networks could help to obtain a more profound insight on the interactions on didactic resources in mathematics among other profiles of citizens. On the other hand, through the use of other keywords, other kinds of interactions could have been found. Future research should investigate more social networks taking into account cultural differences in these debates.

## 5. Conclusions

To improve public health, the need for citizens to know and discuss scientific evidence is being stressed more and more by the scientific literature, in order to be able to make decisions that affect their health with the most information possible. Although this is very clear in the field of health, more efforts need to be made in the educational areas to promote citizen engagement with scientific evidence on education. However, the present study shows an optimistic landscape by evincing that some individuals are debating about mathematics resources in social media, and some are even including the discourse on scientific evidence when talking about these resources. To continue promoting this pathway and engage more citizens in this discourse, researchers and citizens should engage in collective actions to continue engaging citizens with scientific evidence on mathematics. Their engagement in educational scientific evidence would improve the achievement of many students in mathematics.

## Figures and Tables

**Table 1:** Reddit communities used for the extraction.

Reddit Communities	Number of Posts per Community
Community 1	9062
Community 2	9447
Community 3	760
Community 4	983
Community 5	10,000
Community 6	1069
Total	31,321

**Table 2:** Instagram hashtags used for the extraction.

Instagram Hashtags	Number of Posts per Hashtag
qualityeducation	1000
Education	1000
Feminism	1000
Scienceeducation	1000
Stopbullying	1000
Total	5000

**Table 3:** Twitter hashtags used for the extraction.

Twitter Hashtags and Topics	Number of Tweets per Hashtag and Topic
#books	9999
#DigitalDecade	502
#FutureofEurope	1995
Schools	9999
Wikipedia	10,002
#education	10,001
#equality	10,001
#school	10,001
#students	10,000
#publiceducation	255
#EducationInSongOrFilm	2825
#learning	9999
Total	85,579

**Table 4:** Facebook pages used for the extraction.

Facebook Pages	Number of Posts per Page
Page 1	1635
Page 2	651
Page 3	1052
Page 4	644
Page 5	3814
Page 6	7268
Total	15,064

**Table 5:** Number of posts found with each keyword in Reddit, Instagram, Twitter and Facebook.

Keywords	Posts in Reddit	Posts in Instagram	Posts in Twitter	Posts in Facebook	Total
Calculation	21	0	10 (5)	10	41 (36)
Numbers	97 (96)	24 (22)	98 (35)	55 (54)	274 (207)
Numeracy Skills	0	0	0	0	0
Numeracy	1	0	4 (3)	0	5 (4)
Manipulatives	1	0	0	20	21
Resources	195 (180)	68 (67)	569 (156)	76 (68)	908 (471)
Math	384	127 (118)	1555 (312)	240 (239)	2306 (1062)
Total	669 (661)	219 (202)	2236 (511)	401 (381)	3555 (1755)

The numbers in brackets represent the repeated posts (reposts or posts that appear in two or more keywords).

**Table 6:** Number of posts related to didactic resources for mathematics per category in each social network analyzed.

	Reddit	Instagram	Twitter	Facebook	Total
Not scientific evidence	41	8	21	133	203
Certified scientific evidence	0	0	0	2	2
Supposed scientific evidence	2	0	2	4	8
Total	43	8	23	139	213

## Data Availability

The data presented in this study are openly available in Zenodo at [https://doi.org/10.5281/zenodo.4729725].

## References

[1] Racionero S., Padrós M. (2010). The Dialogic Turn in Educational Psychology. El Giro Dialógico En La Psicología de La Educación. Rev. Psicodidáctica.

[2] Flecha R. (2020). Contributions from Social Theory to Sustainability for All. Sustainability.

[3] Flecha R., van den Besselaar R., Flecha A., Radauer P. (2018). Societal Impact. Monitoring the Impact of EU Framework Programmes.

[4] European Commission (2018). A New Horizon for Europe: Impact Assessment of the 9th EU Framework Programme for Research and Innovation.

[5] Soler M., Gómez A. (2020). A Citizen’s Claim: Science with and for Society. Qual. Inq..

[6] Torras-Gómez E., Guo M., Ramis M. (2019). Sociological Theory from Dialogic Democracy. Int. Multidiscip. J. Soc. Sci..

[7] Aiello E., Donovan C., Duque E., Fabrizio S., Flecha R., Holm P., Molina S., Oliver E., Reale E. (2020). Effective Strategies That Enhance the Social Impact of Social Sciences and Humanities Research. Policy Press.

[8] Pulido C.M., Redondo-Sama G., Sordé-Martí T., Flecha R. (2018). Social Impact in Social Media: A New Method to Evaluate the Social Impact of Research. PLoS ONE.

[9] Van der Tempel J., Noormohamed A., Schwartz R., Norman C., Malas M., Zawertailo L. (2016). Vape, Quit, Tweet? Electronic Cigarettes and Smoking Cessation on Twitter. Int. J. Public Health.

[10] Dredze M., Broniatowski D.A., Smith M.C., Hilyard K.M. (2016). Understanding Vaccine Refusal: Why We Need Social Media Now. Am. J. Prev. Med..

[11] Rovetta A., Bhagavathula A.S. (2020). COVID-19-Related Web Search Behaviors and Infodemic Attitudes in Italy: Infodemiological Study. JMIR Public Health Surveill..

[12] Pulido C.M., Villarejo-Carballido B., Redondo-Sama G., Gómez A. (2020). COVID-19 Infodemic: More Retweets for Science-Based Information on Coronavirus than for False Information. Int. Sociol..

[13] Pulido C.M., Ruiz-Eugenio L., Redondo-Sama G., Villarejo-Carballido B. (2020). A New Application of Social Impact in Social Media for Overcoming Fake News in Health. Int. J. Environ. Res. Public Health.

[14] Vraga E.K., Bode L. (2018). I Do Not Believe You: How Providing a Source Corrects Health Misperceptions across Social Media Platforms. Inf. Commun. Soc..

[15] Remillard J.T., Van Steenbrugge H., Machalow R., Koljonen T., Krzywacki H., Condon L., Hemmi K. (2021). Elementary Teachers’ Reflections on Their Use of Digital Instructional Resources in Four Educational Contexts: Belgium, Finland, Sweden, and U.S. ZDM Math. Educ..

[16] Knake K.T., Chen Z., Yang X., Tait J. (2021). Pinterest Curation and Student Achievement: The Effects of Elementary Mathematics Resources on Students’ Learning over Time. Elem. Sch. J..

[17] Liljekvist Y.E., Randahl A.-C., van Bommel J., Olin-Scheller C. (2021). Facebook for Professional Development: Pedagogical Content Knowledge in the Centre of Teachers’ Online Communities. Scand. J. Educ. Res..

[18] Vázquez A.R., Hache C. (2019). Language practices in the maths clasroom, an experience online teaching development for teachers of mathematics. J. Res. Math. Educ..

[19] Díez-Palomar J., de Sanmamed A.F.F., García-Carrión R., Molina-Roldán S. (2018). Pathways to Equitable and Sustainable Education through the Inclusion of Roma Students in Learning Mathematics. Sustain. Sci. Pract. Policy.

[20] Díez-Palomar J., Olivé J.C. (2015). Using Dialogic Talk to Teach Mathematics: The Case of Interactive Groups. ZDM.

[21] Gómez A., Padrós M., Ríos O., Mara L.C., Pukepuke T. (2019). Reaching Social Impact through Communicative Methodology. Researching with rather than on Vulnerable Populations: The Roma Case. Front. Educ..

[22] Redondo-Sama G., Díez-Palomar J., Campdepadrós R., Morlà-Folch T. (2020). Communicative Methodology: Contributions to Social Impact Assessment in Psychological Research. Front. Psychol..

[23] Gómez A., Puigvert L., Flecha R. (2011). Critical Communicative Methodology: Informing Real Social Transformation through Research. Qual. Inq..

[24] Cabré-Olivé J., Flecha-García R., Ionescu V., Pulido C., Sordé-Martí T. (2017). Identifying the Relevance of Research Goals through Collecting Citizens’ Voices on Social Media. Int. Multidiscip. J. Soc. Sci..

[25] Most Used Social Media 2021. https://www.statista.com/statistics/272014/global-social-networks-ranked-by-number-of-users/.

